# Patients with chronic cluster headache may show reduced activity energy expenditure on ambulatory wrist actigraphy recordings during daytime attacks

**DOI:** 10.1002/brb3.3360

**Published:** 2024-01-02

**Authors:** Nicolas Vandenbussche, Jonas Van Der Donckt, Mathias De Brouwer, Bram Steenwinckel, Marija Stojchevska, Femke Ongenae, Sofie Van Hoecke, Koen Paemeleire

**Affiliations:** ^1^ Department of Neurology Ghent University Hospital Ghent Belgium; ^2^ Department of Basic and Applied Medical Sciences, Faculty of Medicine and Health Sciences Ghent University Ghent Belgium; ^3^ IDLab, Ghent University—imec Ghent Belgium

**Keywords:** accelerometer, cluster headache, digital, movement, wearable

## Abstract

**Objective:**

To investigate the changes in activity energy expenditure (AEE) throughout daytime cluster headache (CH) attacks in patients with chronic CH and to evaluate the usefulness of actigraphy as a digital biomarker of CH attacks.

**Background:**

CH is a primary headache disorder characterized by attacks of severe to very severe unilateral pain (orbital, supraorbital, temporal, or in any combination of these sites), with ipsilateral cranial autonomic symptoms and/or a sense of restlessness or agitation. We hypothesized increased AEE from hyperactivity during attacks measured by actigraphy.

**Methods:**

An observational study including patients with chronic CH was conducted. During 21 days, patients wore an actigraphy device on the nondominant wrist and recorded CH attack‐related data in a dedicated smartphone application. Accelerometer data were used for the calculation of AEE before and during daytime CH attacks that occurred in ambulatory settings, and without restrictions on acute and preventive headache treatment. We compared the activity and movements during the pre‐ictal, ictal, and postictal phases with data from wrist‐worn actigraphy with time‐concordant intervals during non‐headache periods.

**Results:**

Four patients provided 34 attacks, of which 15 attacks met the eligibility criteria for further analysis. In contrast with the initial hypothesis of increased energy expenditure during CH attacks, a decrease in movement was observed during the pre‐ictal phase (30 min before onset to onset) and during the headache phase. A significant decrease (*p* < .01) in the proportion of high‐intensity movement during headache attacks, of which the majority were oxygen‐treated, was observed. This trend was less present for low‐intensity movements.

**Conclusion:**

The unexpected decrease in AEE during the pre‐ictal and headache phase of daytime CH attacks in patients with chronic CH under acute and preventive treatment in ambulatory settings has important implications for future research on wrist actigraphy in CH.

## Graphical abstract

Patients with chronic cluster headache may show reduced activity energy expenditure on ambulatory wrist actigraphy recordings during daytime attacks (Vandenbussche et al. Brain and Behavior). This observational study investigated activity energy expenditure (AEE) throughout daytime cluster headache (CH) attacks in patients with chronic CH. A significant decrease (*p* < .01) in the proportion of high‐intensity movement before and during mostly oxygen‐treated headache attacks was observed. This unexpected decrease in AEE during the pre‐ictal and headache phase of daytime CH attacks in patients with chronic CH under acute and preventive treatment in ambulatory settings has implications for future research on wrist actigraphy in CH.

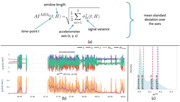

## LIMITATIONS

Limitations to our study should be addressed. First, the low number of patients in our study and the low number of CH attacks registered may have introduced a sampling error and make it hard to draw definitive conclusions. Generalization to other patients with CH is therefore not possible at the moment, especially patients with the episodic form who were not included in this study. The results are preliminary and part of a larger research effort into the detection of behavioral changes of headache patients before, during, and after CH attacks. Second, our study design utilized streaming data, potentially resulting in fewer headaches fulfilling the headache data ratio requirement due to missing data. Böttcher et al. (2022) observed that using the Empatica E4 in a non‐streaming mode generally yields a higher data ratio, but streaming results in a lower user burden as patients do not have to manually download the data from the device and send it to the cloud. Third, we cannot extrapolate our findings immediately to patients with ECH as only CH attacks from patients with longstanding CCH were investigated. Fourth, the study of untreated, and thus more severe CH attacks, most likely yields different data during the headache phase of the CH attack, and preventive treatment may have its influence too. Our real‐world observational study design allowed patients to use all types of CH treatments, both acute and preventive, at their discretion as it was deemed unethical to restrict the use of highly effective treatments at this stage of the research. Fifth, we did not collect any momentary assessments of participants’ behavior during the CH attacks. Therefore, any potential causal factor for hypoactivity (e.g., oxygen mask holding) relies on indirect evidence retrieved from the actigraphy data and user input into the smartphone application.

## 1 INTRODUCTION

Cluster headache (CH) is a primary headache disorder characterized by attacks of severe to very severe unilateral pain which is orbital, supraorbital, temporal, or in any combination of these sites, lasting 15–180 min when untreated (Headache Classification Committee of the International Headache Society [IHS], 2018). The International Classification of Headache Disorder Third Edition (ICHD‐3) criteria for CH furthermore require that CH attacks are associated with ipsilateral cranial autonomic symptoms, for example, lacrimation, and/or a sense of restlessness or agitation (IHS, 2018). Pre‐ictal symptoms were only recently studied in detail and are in fact very common; they include both local and general symptoms (Snoer, Lund, Beske, Hagedorn, et al., 2018, Snoer, Lund, Beske, Jensen, et al., 2018). Patients are diagnosed with episodic cluster headache (ECH) if they have periods of CH attacks that are separated by pain‐free periods lasting at least 3 months, whereas the diagnosis of chronic cluster headache (CCH) is given when CH attacks occur for at least 1 year without a remission period or with remissions lasting less than 3 months (IHS, 2018; Snoer, Lund, Beske, Jensen, et al., 2018).

Patient descriptions and clinical observations indicate that hyperactivity and/or increased movement may be an associated symptom during CH attacks. Ekbom (1970) was the first to report that patients are unable to sit still during a CH attack, this in contrast to migraine patients experiencing increased pain with head movements. Blau (1993) asked patients to act out their behaviors during CH attacks and these included pacing while clutching the head, sitting and rocking backward and forwards while holding the head, pressing the affected eye or temple, and hitting the forehead. Even violent, destructive behavior and self‐inflicted injuries have been described (Blau, 1993; Dodick et al., 1999; Ekbom, 1970; May, 2005). These behaviors seemed to be related to the severity of the pain (Russell, 1981). More recently CH pain has been conceptualized as exteroceptive pain with associated fight–flight response including motor restlessness and agitation, in contrast to migraine pain described as visceral pain (Montagna & Pierangeli, 2010).

Actigraphy is a noninvasive method that utilizes continuous monitoring sensors worn on the body to objectively measure movement and physical activity (Saeed et al., 2019). Therefore, actigraphy may provide insights into patient behaviors during the pre‐ictal, ictal, and postictal phases of CH attacks. The primary objective of our research is to investigate the differences in activity energy expenditure (AEE) during CH attacks using a wrist‐worn actigraphy device in unconstrained ambulatory environments (Hills et al., 2014). In line with the existing medical literature, we hypothesize that AEE measured by wrist actigraphy increases during CH attacks. A secondary objective of the study is to examine AEE changes in the pre‐ictal phase. By hypothesizing that restlessness and agitation can be measured using actigraphy, this technique holds promise as a digital biomarker for identifying CH attacks.

## 2 METHODS

### 2.1 Participants

Patients with a diagnosis of CCH (ICHD‐3 diagnosis 3.1.2), recruited nonconsecutively from the tertiary headache clinic of the Ghent University Hospital, and not suffering from any other headache syndrome (except for infrequent tension‐type headache), were included in the study.

Inclusion criteria were as follows: age between 18 and 65 years, at least 5 CH attacks per week expected (i.e., during intake not in a CH attack‐free period), no significant medical comorbidity interfering with movement and activity, no drug or alcohol abuse, and no use of beta‐blockers. Participants also needed to have regular sleep‐wake rhythms and were not nighttime shift‐workers.

All participants had access to their habitual CH attack treatment of subcutaneous sumatriptan 6 mg or high‐flow oxygen at 12–15 L/min via a non‐rebreathing mask and other acute treatments at their own discretion. There were no restrictions on the use of preventive treatments for CH for the duration of the study.

### 2.2 Wrist‐worn actigraphy sensor and smartphone application

The Empatica E4 device was used for the actigraphy recordings on the nondominant wrist (McCarthy et al., 2016). This device is a CE‐certified medical‐grade wearable that offers physiological data acquisition. It has an internal memory that can store up to 60 h of data, but in this study, the data were sent in real‐time over a Bluetooth Low Energy connection to a smartphone that streamed the data to our cloud. Empatica E4 devices have an onboard microelectromechanical system type 3‐axis accelerometer that measures continuous gravitational force (g) applied to each of the three spatial dimensions (*x*, *y*, and *z*). The scale is set to  ±2 g. These accelerometer signals are collected at 32 Hz for the three dimensions.

**FIGURE 1:**
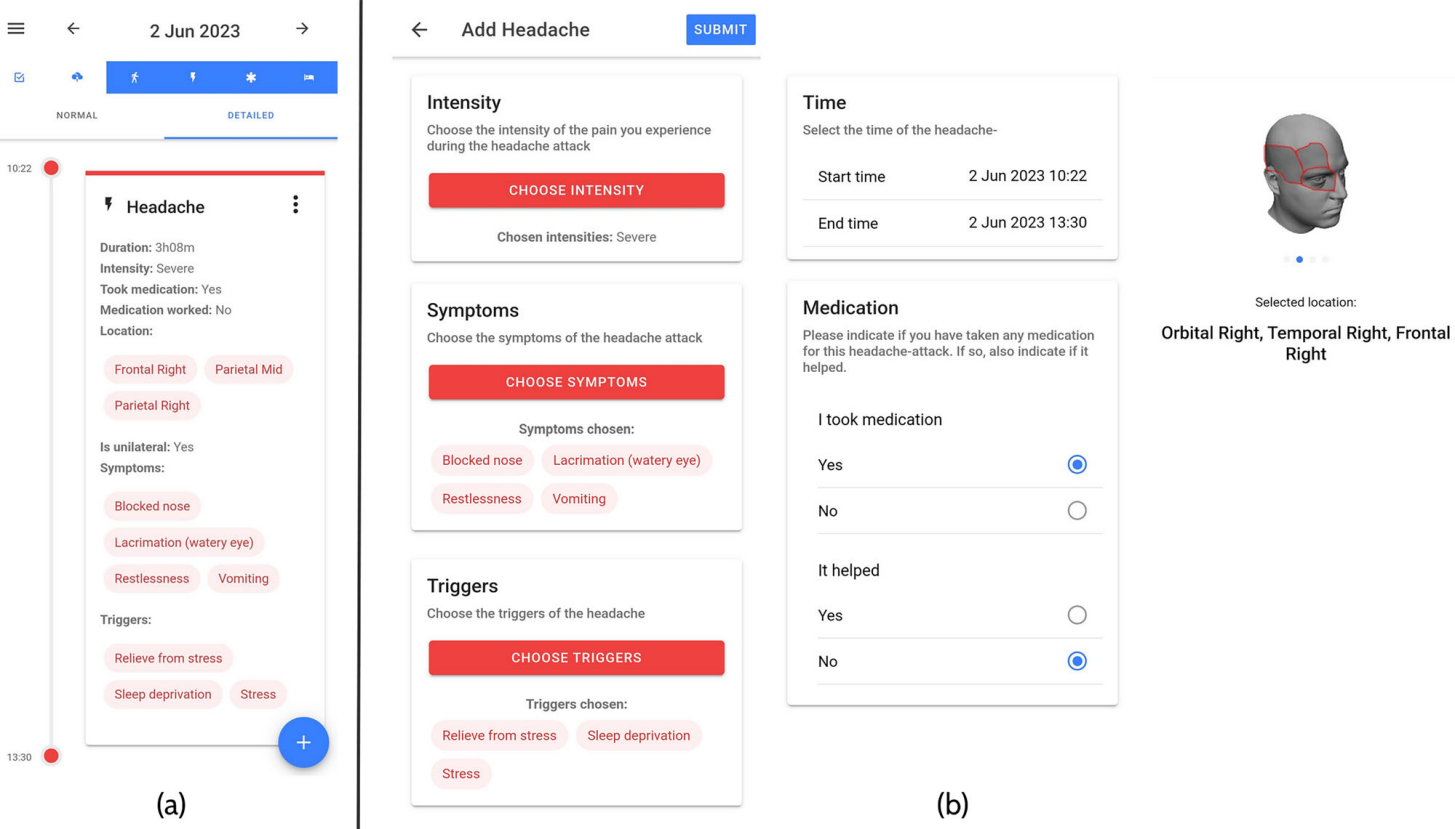
Illustration of “mBrain” app timeline view (a) and corresponding headache registration (b).

Participants manually documented CH attacks using a dedicated headache smartphone application (the “mBrain” app) developed for this study (Figure 1). The recorded CH attack data included the time of onset (hour and minutes) and end (hour and minutes) of each CH attack, the intensity of the CH attack measured on a 5‐point Likert Scale ranging from no pain to very severe pain, the use of CH attack medication and the effectiveness of those treatments (part b of Figure 1) (De Brouwer et al., 2022; Van Der Donckt et al., 2022). Participants were trained during the intake visit to register the details of an experienced CH attack in the application as soon as possible after the CH attack had stopped.

### 2.3 Study design

The study consisted of a baseline study visit and a final study visit 21 days later. During the study period, participants were instructed to wear the device as much as possible (daytime and nighttime) which ensured consistency in data collection and minimized disruption to daily routines. They were also advised to charge the sensor device at least once a day in the evening. Participants were asked to perform their regular daily activities during the measurement period. However, they were asked to take off the Empatica E4 device when engaging in activities involving water (e.g., showering and swimming), heat (e.g., barbecuing), or cold (e.g., working in freezers).

### 2.4 Absolute activity index as a marker for activity energy expenditure

**FIGURE 2:**
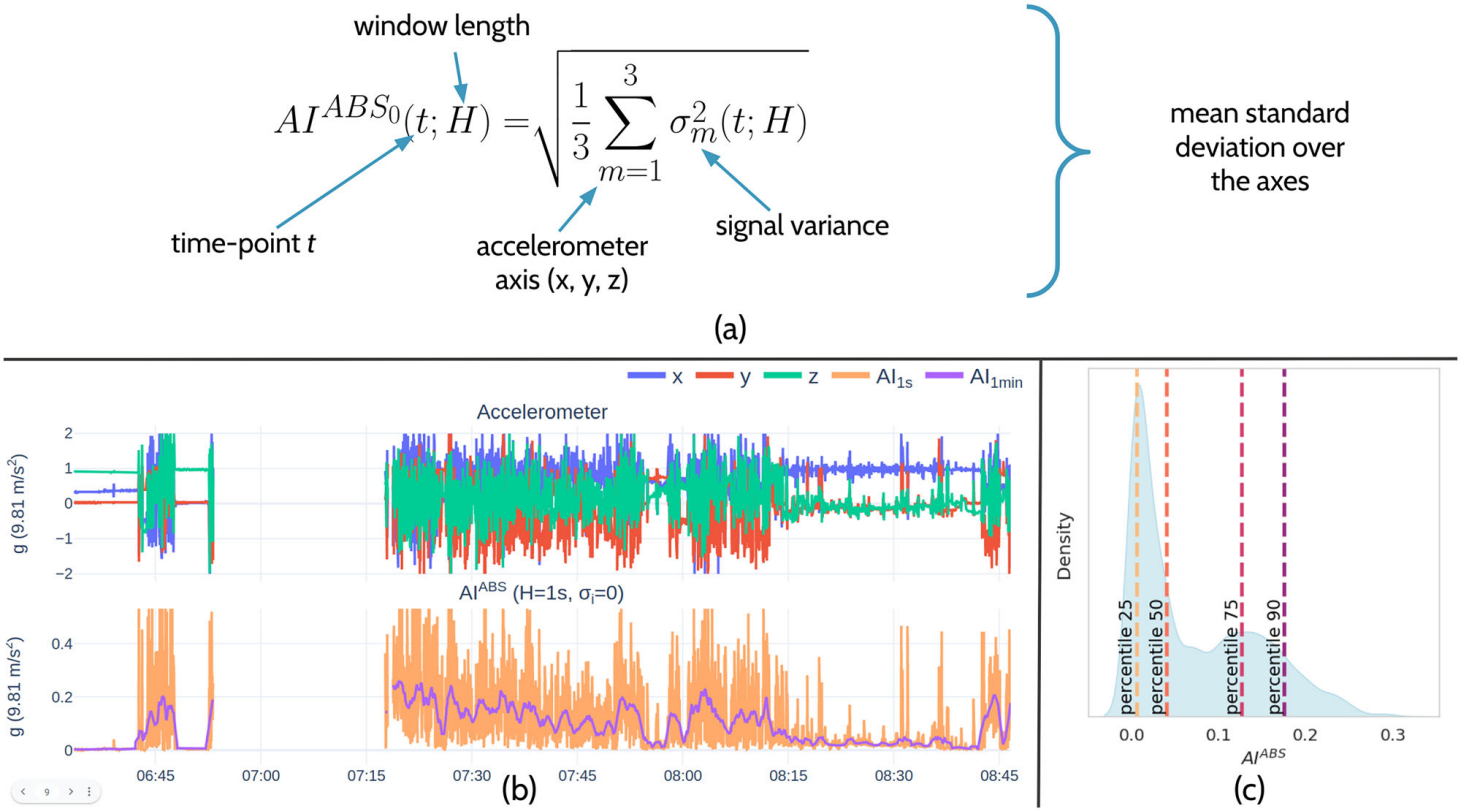
Illustration of AI^ABS^ computation and its distribution properties. To calculate the AI^ABS^, the simplified equation given in (a) was used, which assumes a systematic noise‐variance *σ_i_* of zero. Subplot (b) portrays the 1‐min aggregated AI^ABS^ for an Empatica E4 accelerometer excerpt. Subfigure (c) demonstrates the distribution and percentiles of the AI^ABS^ of (b). *𝙷* = variance window length, *t* = time point, *σ*_m_ = signal variance of wrist acceleration over axis *x*, *y*, or *z*.

From the time series data of g‐force measurements, the absolute activity index (AI^ABS^) is calculated and used as the metric of AEE (Bai et al., 2016). As shown in Figure 2a, the AI^ABS^ is formulated as the square root of the mean variance of the raw accelerometer signals, with the variance computed over a fixed period, 𝙷. The AI^ABS^ exhibits a robust correlation with activity intensity (Bai et al., 2016). Empirical analysis using the Empatica E4 device revealed that the systematic noise variance, *σ_i_*, was negligible, and therefore, *σ_i_* was set to 0 for the calculation of the AI^ABS^, leading to a simplification of the AI^ABS^ formula. In alignment with Bai et al. (2016) the AI^ABS^ is computed over a time‐period 𝙷 of 1 s, corresponding to 32 sampling points. Afterward, also in accordance with Bai et al., these second‐by‐second AI^ABS^ values are aggregated to a per‐minute AI, by averaging the 1‐s AI^ABS^ values within each 1‐min period (Figure 2b). For time intervals of interest, such as the CH attack interval, the AI^ABS^ values can be represented by a distribution. Considering the non‐normal distribution of AI^ABS^ over these intervals, as depicted in Figure 2c, we employ a range of quantile‐based features for analysis. Specifically, we calculate the median AI^ABS^ (i.e., 50th percentile), as well as the 25th, 75th, and 90th percentiles. We included percentile 90 to examine movement associated with higher energy expenditure, in alignment with our hypothesis.

### 2.5 Eligibility criteria for the analysis of CH attacks and corresponding non‐CH intervals

To be eligible for analysis, CH attacks had to fulfill the following criteria. The CH attacks must have occurred during daytime periods without overlap between the headache interval (as documented by the participant during headache registration) and nighttime (between 11:00 p.m. and 7:30 a.m.). Additionally, a minimum data ratio of 75% during CH attacks was used to ensure a reliable analysis of actigraphy data (i.e., no more than 25% of data missing during the CH attack).

For comparison purposes, intervals without CH attacks from the same participant were used, subject to the following conditions. Non‐CH intervals had to be at least 24 h distant from the start and end of CH attacks, and a time range equal to the CH attack was used on the same type of day, either weekdays or during weekends. In cases when multiple eligible non‐CH intervals were identified, these intervals were accumulated to construct a single non‐CH AI^ABS^ distribution to be paired against the corresponding CH attack. Data from Belgian holidays were excluded from the analysis.

### 2.6 Analysis and statistics

Demographic data and participant‐specific information (i.e., age, sex, diagnosis, age of onset, and treatment regimens related to CH) are provided in a descriptive manner as proportions and means with standard deviations (SD). Recorded CH attacks are described with mean and SD of CH attack duration (in minutes), mean and SD of CH attack intensity, the proportion of CH attacks treated with acute therapy, and the proportion of acute‐treated CH attacks which were successfully managed.

The first analysis assesses AEE during CH attacks by using each CH headache interval as time range. Specifically, we calculated AI^ABS^ distributions for eligible single CH attacks and their corresponding non‐CH data. Percentile AI^ABS^ differences (ΔAI^ABS^) for single CH attacks were determined by subtracting the CH attack percentile AI^ABS^ value from the non‐CH data's percentile value. Furthermore, this analysis also assesses the impact of acute treatment type, which is represented via a color hue.

The second analysis examined AEE distributions for specific time intervals relative to headache onset, including 3–1 h before onset, 1 h to 30 min before onset, 30 min to onset, onset to 30 min after, 30 min to 1 h after, 1–2 h after, and 2–3 h after. Percentile AI^ABS^ interval differences were calculated by subtracting the CH attack interval percentiles from the corresponding non‐CH interval percentiles. The number of events may vary for each interval, as the ≥75% data ratio must be fulfilled for both CH and non‐CH intervals.

Given the high likelihood of non‐normality in the separate AI^ABS^ interval distributions as depicted by Figure 2c, the Wilcoxon signed‐rank test was employed to evaluate the statistical significance of AI^ABS^ percentile pairs for CH and their corresponding non‐CH intervals. A two‐tailed test was used for each of those analyses. Afterward, all results were subjected to Bonferroni correction for multiple testing; therefore, only *p*‐values corrected for multiple testing are presented.

Due to the exploratory nature of our pilot study and the lack of previous data, no formal sample size calculation was performed before the onset of the study.

All data processing and transformations were conducted via Python 3.8. Statistical testing was performed using the SciPy package (Virtanen et al., 2020). For exploratory data analysis of the raw wearable data, the plotly‐resampler tool was used (Van der Donckt et al., 2022a). Via this exploratory analysis, we assessed whether the time‐ranges of the headache intervals overlapped with typical sleep periods. Using the 11 p.m.–7:30 a.m. nighttime filter, we observed that none of the remaining daytime CH intervals fell within the typical sleep periods of all participants. During the exploratory analysis of the raw wearable data, off‐body periods were observed, which refer to intervals where wearable data were present despite the device not being worn (Berger et al., 2008). To address this issue, an on‐body detection algorithm was used to identify and exclude off‐body periods (Böttcher et al., 2022). This processing step was performed prior to computing the interval data ratios and the AI^ABS^. In order to efficiently compute the AI^ABS^, NumPy functions were leveraged through the tsflex library (Harris et al., 2020; Van der Donckt et al., 2022b).

### 2.7 Ethics

This study was approved by the Committee for Medical Ethics of the Ghent University Hospital (internal ID BC‐07403, approved June 12, 2020). Patients were fully informed on all the aspects of the study (duration, procedures, study visit, etc.) and gave written informed consent at the beginning of the study. Participants received a pseudonymized code throughout the study. Only physician–researchers had the key to decode the participant if required.

## 3 RESULTS

### 3.1 Participants and CH attacks

Four male participants with CCH were included in the analysis (Table 1). All participants had access to high‐flow oxygen and/or sumatriptan 6 mg SC injection for the acute treatment of CH attacks. All participants (*N* = 4) used preventive treatment with verapamil, and two participants used melatonin before bedtime. During the intake discussion, all participants (*N* = 4) reported experiencing restlessness and/or agitation and characterized the pain during typical CH attacks as “severe” to “very severe.”

**TABLE 1:** Descriptive characteristics of participants (*N* = 4).

Patient	Diagnosis	Age	Sex	Age at onset CH	Acute treatment	Preventive treatment	Total CH attacks/daytime/… & > = 75% data/… & eligible non‐CH data
1	CCH	50	Male	36	Oxygen, sumatriptan SC 6 mg	Verapamil 240 mg daily dose	5/3/3/3
2	CCH	64	Male	60	Oxygen, sumatriptan SC 6 mg	Verapamil 480 mg daily dose	14/9/6/6
3	CCH	43	Male	36	Oxygen, sumatriptan SC 6 mg	Verapamil 720 mg daily dose, melatonin 3 mg before bedtime	7/1/1/0
4	CCH	27	Male	15	Oxygen, sumatriptan SC 6 mg, zolmitriptan nasal spray 5 mg, paracetamol 250 mg/acetylsalicylic acid 250 mg/caffeine 65 mg	Verapamil 640 mg daily dose, melatonin 5 mg before bedtime	8/8/6/6

Abbreviations: CCH, chronic cluster headache; CH, cluster headache; mg, milligram; SC, subcutaneous.

In total, 34 CH attacks were recorded. Fifteen of these attacks from three participants met the daytime and data ratio requirements, while also having eligible non‐headache interval(s), and were used for further analysis (seven out of seven CH attacks from one participant could not be used due to six nighttime CH attacks and one CH attack not having an eligible non‐CH interval). The mean duration of the analyzed CH attacks was 37 min (SD 25 min) (see Appendix 1). Thirteen out of the 15 CH attacks (87%) were treated with acute therapy: 8 with high‐flow oxygen, 2 with combination analgesics paracetamol 250 mg/acetylsalicylic acid 250 mg/caffeine 65 mg, 1 with combination high‐flow oxygen and zolmitriptan 5 mg nasal spray, and 2 with non‐specified treatments. Twelve of these 13 CH attacks were found to be treated effectively by the participants (92%) (Table 2). The mean amount of data ratio per individual CH attack was 98.0% (SD 6%). For every analyzed CH attack, the corresponding volume of eligible non‐CH data was equal to or higher than the duration of the CH attack itself.

**TABLE 2:** Descriptive analysis of all cluster headache attacks registered during study.

Description	*N*	Mean duration (SD), minutes	Mean intensity (SD)^a^	Acute treatment use (%)/(*n*/*N*)	Acute treatment effectiveness (%)/(*n*/*N*)
All headaches	34	35 (22)	2.9 (.7)	82% (28/34)	96% (27/28)
Nighttime headaches	13	32 (21)	3.3 (.7)	69% (9/13)	100% (9/9)
Daytime headaches	21	37 (23)	2.6 (.7)	90% (19/21)	95% (18/19)
Daytime headaches w/≥75% data during CH attack interval	16	38 (25)	2.6 (.7)	88% (14/16)	93% (13/14)
Daytime headaches w/≥75% data during CH attack interval and eligible non CH attack interval data	15	38 (26)	2.5 (0.6)	87% (13/15)	92% (12/13)

Abbreviations: *n*/*N*, number; SD, standard deviation.

^a^ Intensity levels: 1 = no pain, 2 = light pain, 3 = moderate pain, 4 = severe pain, 5 = very severe pain.

### 3.2 Activity energy expenditure during CH attacks and by acute treatment type

**FIGURE 3:**
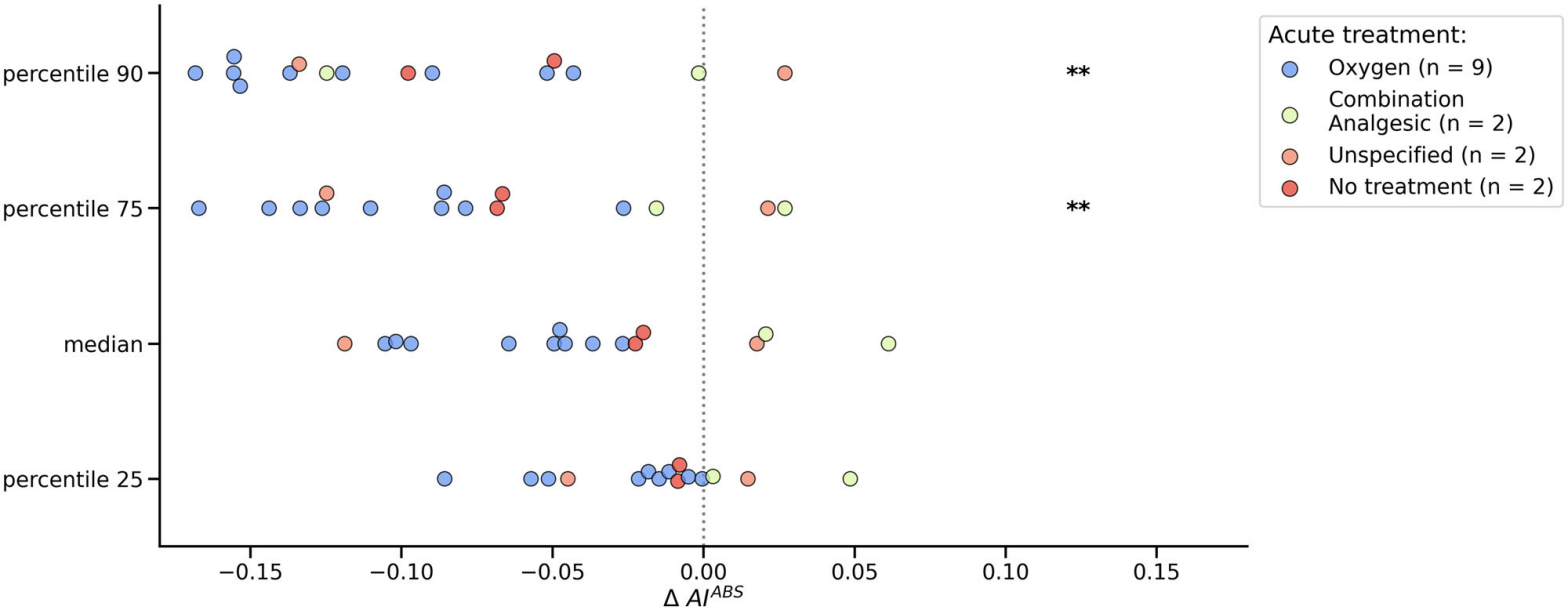
Differences in percentile AI^ABS^ (ΔAI^ABS^) during individual CH attacks and acute treatment type. ΔAI^ABS^ is determined by subtracting the AI^ABS^ percentile value of each CH attack from its corresponding eligible non‐CH value (*N* = 15). Each dot represents the difference for a single CH attack. The acute treatment is represented via a color hue. The treatment types include: no treatment (*N* = 2), unspecified (*N* = 2), oxygen (*N* = 9), and combination analgesic (*N* = 2). An “unspecified” treatment indicates that the participant reported using medication during the headache event, but no specific medication event was reported afterward. AI^ABS^ = absolute activity index, ΔAI^ABS^ = difference between absolute activity indices. Levels of statistical significance (after Bonferroni correction for multiple testing): *p < .05; **p < .01; ***p < .001.

Percentile ΔAI^ABS^ values were calculated for the headache intervals of the 15 qualifying CH attacks, as illustrated in Figure 3. When compared to the corresponding non‐headache intervals, the AI^ABS^ values during CH attacks show a negative ΔAI^ABS^ trend, indicating a reduction in AEE. This reduction is also represented by a significantly lower 75th percentile (*p* = .007) and 90th percentile (*p* = .0014). No significant differences in AI^ABS^ values were found for the 25th percentile (*p* = .205) and median (*p* = .054) between CH attacks and non‐headache intervals) (see Appendix 2). Figure 3 also uses color hues to denote the acute treatment type used during a single CH attack. For all four percentiles, all ΔAI^ABS^ values are negative for the CH attacks that were treated with oxygen, indicating no increases in AEE for all of the nine oxygen‐treated CH attacks. Appendix 3 presents the statistical significance results exclusively for the oxygen treatment group (*N* = 9).

### 3.3 Temporal patterns of activity energy expenditure before and during CH attacks

**FIGURE 4:**
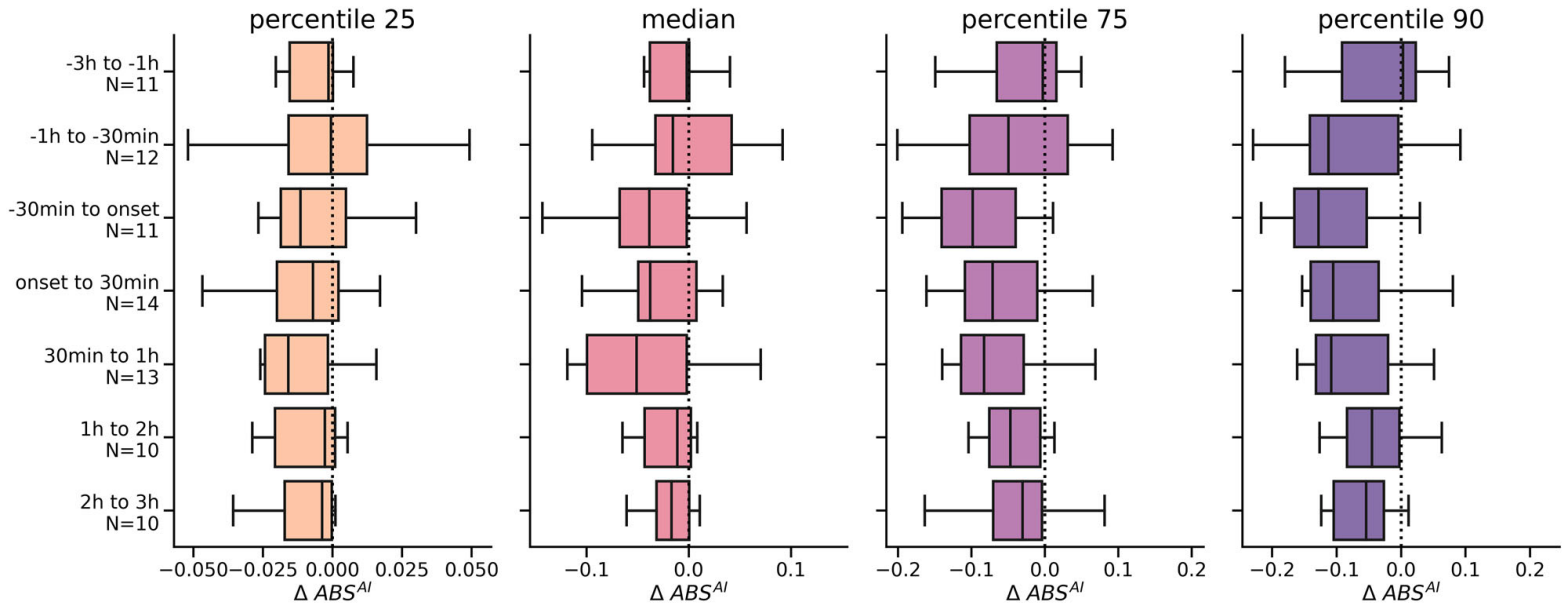
Percentile AI^ABS^ differences (ΔAI^ABS^ distributions) for time ranges relative to the headache onsets. The *y*‐axis labels indicate the number of CH attacks considered, which varies due to the ≥75% data ratio requirement for both CH attack intervals and non‐CH attack intervals. AI^ABS^ = absolute activity index, ΔAI^ABS^ = difference between absolute activity indices, CH = cluster headache, h = hour, N = number. Levels of statistical significance (after Bonferroni correction for multiple testing): *p < .05; **p < .01; ***p < .001.

The decrease of intense movements during headaches, resulting in negative ΔAI^ABS^ values, is observed in Figure 3. This decrease is also reflected in Figure 4 by the negative yet nonsignificant Δ trend of the “onset to 30 min” interval (*N* = 14) for the 75th (*p* = .215) and 90th percentile (*p* = .098). Additionally, Figure 4 shows that this negative ΔAI^ABS^ trend is already observed during the pre‐ictal phase, specifically for the “−1 h to −30 min” (*N* = 12) and “−30 min to onset” (*N* = 11) intervals at the 75th and 90th percentiles. The “30 min to 1 h” (*N* = 13) interval also exhibits a decreasing, but nonsignificant, trend. This negative ΔAI^ABS^ trend persists up to 3 h after onset. No statistically significant differences were observed for any of the interval percentiles, which could be attributed to the reduced sample size of the intervals (see Appendix 4). Analysis for the postictal phase can be found in Appendix 5.

## 4 DISCUSSION

To our knowledge, this is the first objective, real‐world study utilizing wrist actigraphy to measure the AEE before and during CH attacks in patients with CCH during daytime in the ambulatory setting. Actigraphy in the field of CH has only been used in sleep studies so far (Della Marca et al., 2006; Lund et al., 2019). Wearable technology has evolved into a low‐cost continuous measuring modality providing accurate measurements of activity, movement, and energy expenditure. The technology opens up the potential to more accurately research the behavior of patients in the ictal and interictal phases of headache disorders and holds potential as a digital biomarker for CH attacks.

Our real‐world objective actigraphic data challenge the stereotypical idea of behavior during CH attacks in patients using acute treatment. To summarize, our analysis suggests that patients with CCH may show reduced AEE and reduced presence of high‐intensity movements not only during the ictal phase but also during the pre‐ictal phase and potentially the postdromal phase of CH attacks.

Senses of restlessness and/or agitation are common symptoms of (untreated) CH attacks, hence their integration in the ICHD‐3 diagnostic criteria for CH (IHS, 2018; Torelli & Manzoni, 2005). Almost 70%–90% of patients with CH described typical signs of psychomotor agitation (restlessness) during the CH attack in prospective studies (Torelli & Manzoni, 2003). A prospective clinical survey study in patients with CH found 93% of participants reporting restlessness during the CH attacks or that movement did not exacerbate the pain (Bahra et al., 2002). Another prospective survey study found that 88.1% of participants with CH exhibited signs of psychomotor agitation (restlessness) with an inability to keep still or performing different types of actions during typical and untreated CH attacks (Torelli & Manzoni, 2003). Previous scientific reports on the clinical features also documented the sense of restlessness or even compulsed movement as a prominent feature of CH (Blau, 1993; Kudrow & Dalessio, 1981; Manzoni et al., 1983; Mathew, 1997; Torelli & Manzoni, 2005). Restlessness or agitation as an associated symptom may even be triggered with calcitonin gene‐related peptide or nitroglycerin in laboratory‐induced CH attacks (Vollesen et al., 2018; Wei & Goadsby, 2021). Therefore, restlessness is a highly sensitive and highly specific parameter for CH (Torelli & Manzoni, 2005). Of note that multiple studies in Asia (including in Taiwan, Japan, and Korea) have reported on lower percentages of feelings of restlessness and uncoupling from restless behavior (Imai et al., 2011; Ko et al., 2021; Moon et al., 2017).

We infer multiple reasons for our findings of pre‐ictal and ictal hypoactivity which, in turn, create new hypotheses and questions for future research. First, based on the data, the behavioral effects of ictal treatment could force the patient into hypoactivity. This seems especially true for oxygen‐treated CH attacks in our dataset, which can be observed in Figure 3 and Appendix 3. Due to the specific context of the wrist actigraphy and despite being worn on the nondominant wrist, it is possible that patients keep their hand and wrist in a more fixed position, for example, to hold the oxygen mask or holding their heads with their hands during the CH attacks. It can be speculated that these actions might limit intense hands or body movements. It is plausible that chest‐ or hip‐worn actigraphy devices yield different results due to the difference between axial and appendicular motions. Second, although the ICHD‐3 criteria require severe to very severe pain for untreated CH attacks, patients in our study reported the severity of the CH attack as moderate, which may affect behavior (see Table 2). In our study, no restrictions were imposed on acute treatment. Hence, the capability to manage CH attacks, and the high treatment efficacy, could account for both the moderate CH attack intensity and reduction in AEE. Indeed, patients with CCH may experience within‐individual variability in attack severity with attacks of mild‐to‐moderate intensity as reported previously by a Danish group (Hagedorn et al., 2019). Third, from analyzing the literature, the results may not be completely surprising as previous research efforts may have hinted at reduced AEE in relation to certain CH attacks. Snoer et al. analyzed the presence of symptoms in the pre‐ictal, ictal, and postictal phase of CH attacks in a prospective, observational questionnaire‐based study. Apart from 54% of CH attacks having restlessness as a general symptom, also 30.4% of CH attacks were accompanied by decreased energy levels (Snoer, Lund, Beske, Hagedorn, et al., 2018). The authors also found that the most frequent general symptom in the pre‐ictal phase was a sense of restlessness (22.2% of CH attacks), but also that 13.2% of CH attacks had decreased energy levels and 5.6% of CH attacks had fatigue as a pre‐ictal symptom (Snoer, Lund, Beske, Hagedorn, et al., 2018). Snoer et al. have also provided good evidence that general symptoms within the pre‐ictal phase of CH attacks occurred at a median of 20 min prior to the CH attack, in line with our findings of the most significant drop in AEE between 30 min before onset and onset. Furthermore, this Danish study also found “decreased energy levels” and/or “fatigue” as possible pre‐ictal and ictal symptoms in their cohort. Furthermore, for the pre‐ictal phase, Blau and Engel already reported in 1998 that several patients may feel “tired,” “low,” “apathetic,” “listless,” “withdrawn,” “quiet,” or “ill” in the pre‐ictal phase (Blau & Engel, 1998).

We have no doubt that the pain from CH attacks can indeed be excruciating, may lead to a sense of restlessness or agitation, and therefore result in a tendency to pace or move intensely in contrast to the ictal behavior of patients during migraine attacks. This has been documented for many years by patients, clinical experts, and researchers. We are, however, convinced of the quality of our measured data points, the registrations in the headache applications performed by participants, and the data analysis. Our data, however, suggest that, in real‐world settings with unrestricted access to highly efficacious acute treatment for CH attacks, caution is needed when interpreting increased AEE calculated from wrist actigraphy as this may lead to incorrect alarms, misleading results, or over‐detection (Cohen et al., 2009; Sumatriptan Cluster Headache Study, 1991). In the age of rapid access to actigraphy devices, artificial intelligence, and machine learning algorithms, this feature should be addressed carefully when designing data‐driven CH detection models for real‐world use.

## 5 CONCLUSION

Our findings show that wrist actigraphy for the detection of CH attacks in ambulatory environments with no restrictions on acute treatment may measure reduced activity during CH attacks, in contrast to our initial assumption that patients would show increased AEE due to movement during CH attacks. Our data may provide a methodological and hypothesis‐generating basis for future real‐world actigraphy studies in a larger sample of patients with CH. Further research is required to determine the use of wrist actigraphy for the analysis of movement during CH attacks and as a digital biomarker for CH.

## AUTHOR CONTRIBUTIONS

**Nicolas Vandenbussche**: Conceptualization; investigation; formal analysis; writing—original draft; writing—review and editing; methodology. **Jonas Van Der Donckt**: Conceptualization; formal analysis; writing—original draft; writing—review and editing; visualization; methodology. **Mathias De Brouwer**: Conceptualization; writing—review and editing; methodology. **Bram Steenwinckel**: Conceptualization; writing—review and editing; methodology. **Marija Stojchevska**: Conceptualization; writing—review and editing; methodology. **Femke Ongenae**: Conceptualization; writing—review and editing; supervision; methodology. **Sofie Van Hoecke**: Conceptualization; writing—review and editing; supervision; methodology. **Koen Paemeleire**: Conceptualization; writing—review and editing; supervision; methodology.

## CONFLICT OF INTEREST STATEMENT

Nicolas Vandenbussche has received travel grants and consulting fees from Novartis AG, TEVA Pharmaceuticals Industries Ltd., AbbVie/Allergan and Pfizer Inc. Koen Paemeleire has received personal compensation from AbbVie/Allergan, Amgen/Novartis AG, Eli Lilly and Company, Lundbeck, Pfizer, Teva Pharmaceuticals Industries Ltd., and Man & Science for consulting, serving on a scientific advisory board, and/or speaking; he is/was a clinical trial investigator for Almirall (almotriptan), Amgen/Novartis AG (erenumab), Eli Lilly and Company (galcanezumab, lasmiditan), Lundbeck (eptinezumab), and Autonomic Technologies Inc. (sphenopalatine ganglion stimulation). Jonas Van Der Donckt, Mathias De Brouwer, Bram Steenwinckel, Marija Stojchevska, Femke Ongenae, and Sofie Van Hoecke report no conflicts of interest.

### PEER REVIEW

The peer review history for this article is available at https://publons.com/publon/10.1002/brb3.3360.

## CLINICAL TRIALS REGISTRATION NUMBER

NCT04949204 (www.ClinicalTrials.gov).

## Supporting information

Supporting Information

## Data Availability

The data that support the findings of this study are available on request from the corresponding author. The data are not publicly available due to privacy or ethical restrictions.
