# Supplementary material for: Patients with chronic cluster headache may show reduced activity energy expenditure on ambulatory wrist actigraphy recordings during daytime attacks
Source: Brain Behav. 2024 Jan 2;14(1):e3360. doi: 10.1002/brb3.3360 (PMC10761329; doi:10.1002/brb3.3360)

**Appendix 1:**

**Table: Descriptive analysis of headache duration.**

| **description** | **N** | mean+/-std | min | 25% | 50% | 75% | max |
| --- | --- | --- | --- | --- | --- | --- | --- |
| All headaches | 34 | 35min+/-22min | 8min | 20min | 30min | 47min | 1h42min |
| Nighttime headaches | 13 | 32min+/-23min | 9min | 15min | 29min | 42min | 1h25min |
| Daytime headaches | 21 | 37min+/-23min | 8min | 21min | 31min | 50min | 1h42min |
| Daytime headaches w/ ≥75% data during | 16 | 38min+/-25min | 8min | 21min | 31min | 50min | 1h42min |
| Daytime headaches w/ ≥75% data during & eligible non CH data | 15 | 38min+/-26min | 8min | 20min | 31min | 51min | 1h42min |

**Appendix 2:**

**Table: Accompanying p-values of whole headache ΔAI^ABS^** **(N=15, Figure 3 - all AI^ABS^** **pairs).**

| **Percentile** | **p-value** | **Corrected p value** |
| --- | --- | --- |
| Percentile 25 | 0.026 | 0.205 |
| Median | 0.007 | 0.054 |
| Percentile 75 | 8.5e-4 | *0.007*** |
| Percentile 90 | 1.8e-4 | *1.4e-3*** |

***Note****:* * = p < 0.05, ** is p < 0.1, *** is p < 0.001

**Appendix 3:**

**Table: Accompanying p-values of whole headache ΔAI^ABS^** **for oxygen-acute-treated CH attacks (N=9, Figure 3 - oxygen treatment group).**

| **Percentile** | **p-value** | **Corrected p value** |
| --- | --- | --- |
| Percentile 25 | 0.004 | *0.031** |
| Median | 0.004 | *0.031** |
| Percentile 75 | 0.004 | *0.031** |
| Percentile 90 | 0.004 | *0.032** |

***Note****:* * = p < 0.05, ** is p < 0.1, *** is p < 0.001

**Appendix 4:**

**Table: Accompanying p-values of ΔAI^ABS^ intervals relative to headache onset (Figure 4).**

| **Time interval** | **Percentile** | **p-value** | **Corrected p-value** |
| --- | --- | --- | --- |
| -3h to -1h (N=11) | Percentile 25 | 0.123 | 1 |
|  | Median | 0.123 | 1 |
|  | Percentile 75 | 0.206 | 1 |
|  | Percentile 90 | 0.413 | 1 |
| -1h to -30min (N=12) | Percentile 25 | 1 | 1 |
|  | Median | 0.791 | 1 |
|  | Percentile 75 | 0.129 | 1 |
|  | Percentile 90 | 0.021 | 0.672 |
| -30min to onset (N=11) | Percentile 25 | 0.464 | 1 |
|  | Median | 0.083 | 1 |
|  | Percentile 75 | 0.002 | 0.063 |
|  | Percentile 90 | 0.005 | 0.156 |
| Onset to 30 min (N=14) | Percentile 25 | 0.194 | 1 |
|  | Median | 0.024 | 0.785 |
|  | Percentile 75 | 0.007 | 0.215 |
|  | Percentile 90 | 0.003 | 0.098 |
| 30 min to 1h (N=13) | Percentile 25 | 0.068 | 1 |
|  | Median | 0.032 | 1 |
|  | Percentile 75 | 0.010 | 0.336 |
|  | Percentile 90 | 0.005 | 0.148 |
| 1h to 2h (N=10) | Percentile 25 | 0.322 | 1 |
|  | Median | 0.275 | 1 |
|  | Percentile 75 | 0.160 | 1 |
|  | Percentile 90 | 0.064 | 1 |
| 2 to 3h (N=10) | Percentile 25 | 0.131 | 1 |
|  | Median | 0.193 | 1 |
|  | Percentile 75 | 0.131 | 1 |
|  | Percentile 90 | 0.048 | 1 |

***Note****:* * = p < 0.05, ** is p < 0.1, *** is p < 0.001

**Appendix 5:**

**Figure: Headache intervals relative to CH end.** The y-axis labels indicate the number of attacks considered, which varies due to the ≥75% data ratio requirement for both CH and non-CH intervals. *Abbreviations*: AI^ABS^ = absolute activity index, ΔAI^ABS^ = difference between absolute activity indices, CH = cluster headache, h = hour, N = number. Levels of statistical significance (after Bonferroni correction for multiple testing): * p < 0.05; ** p < 0.01; *** p < 0.001.


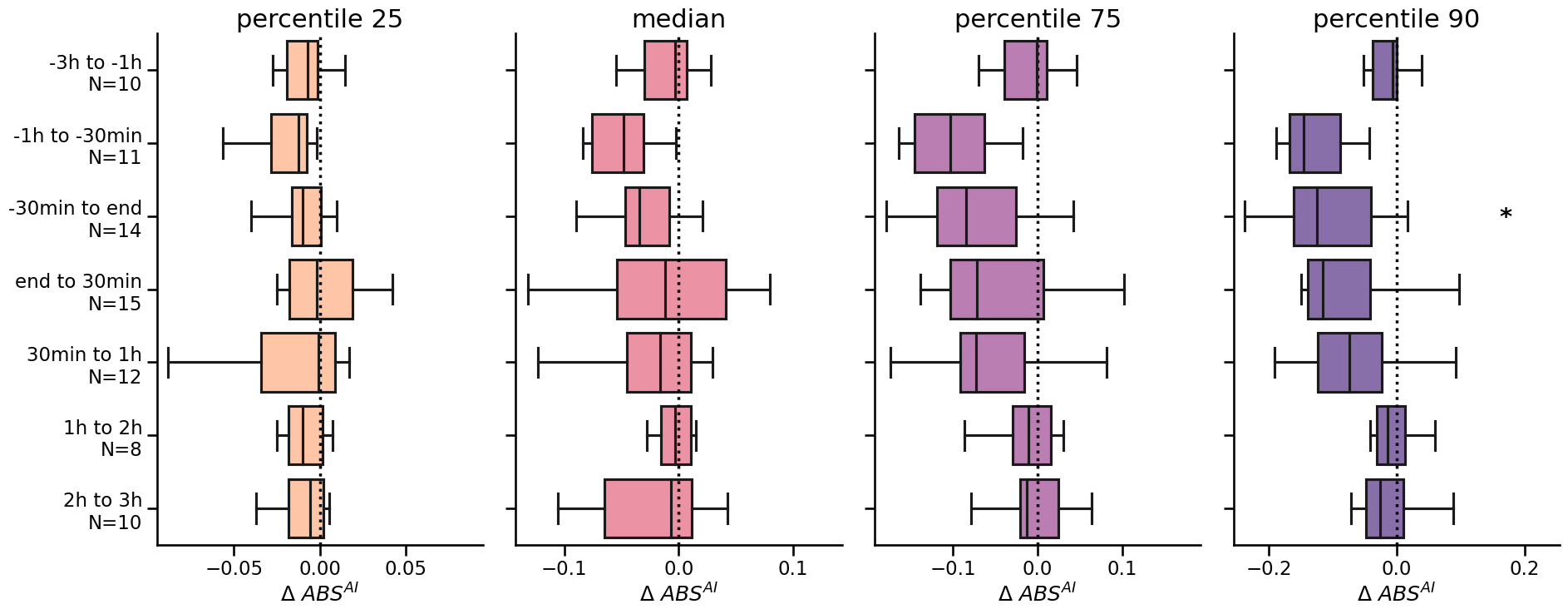

Supplement: Supplementary file 1 — Supporting Information [file BRB3-14-e3360-s001.docx]
